# Real-world treatment patterns and effectiveness among patients with metastatic colorectal cancer treated with ziv-aflibercept in community oncology practices in the USA

**DOI:** 10.1007/s12032-017-1049-4

**Published:** 2017-11-04

**Authors:** Jasmina I. Ivanova, Kimberly R. Saverno, Jennifer Sung, Mei Sheng Duh, Chen Zhao, Sean Cai, Francis Vekeman, Aaron Peevyhouse, Ravinder Dhawan, Charles S. Fuchs

**Affiliations:** 1 Analysis Group, Inc., 10 Rockefeller Plaza, 15th Floor, New York, NY 10020 USA; 2 Vector Oncology, Memphis, TN USA; 3 Sanofi US, Bridgewater, NJ USA; 4 Analysis Group, Inc., Boston, MA USA; 5 Analysis Group, Inc., Montreal, QC Canada; 6 Dana-Farber Cancer Institute and Harvard Medical School, Boston, MA USA

**Keywords:** mCRC, Colorectal cancer, Ziv-aflibercept, Experience, Outcomes, Clinical practice, Post-oxaliplatin, Second line

## Abstract

Routine clinical practice data often differ from clinical trials. This study describes real-world treatment patterns and effectiveness among patients with metastatic colorectal cancer (mCRC) receiving ziv-aflibercept in non-academic, community oncology practices in the USA. De-identified electronic medical records from Vector Oncology and Altos Solutions databases were analysed. We identified 218 patients diagnosed with mCRC who had received prior oxaliplatin therapy and initiated ziv-aflibercept as part of second-line or later-line therapy. Overall survival (OS) and progression-free survival (PFS) were estimated using Kaplan–Meier analysis. Mean age was 62.8 years at ziv-aflibercept initiation. Most patients (91.7%) received bevacizumab before ziv-aflibercept, 95.4% initiated ziv-aflibercept with FOLFIRI or another irinotecan-based regimen, and 59.6% had received prior irinotecan. Overall, 24.8% of patients initiated ziv-aflibercept in second line, 31.7% in third line, 21.6% in fourth line and 22.0% in later lines of therapy. Mean duration of ziv-aflibercept treatment was 5.3 months. For patients initiating ziv-aflibercept in second-, third- and fourth-line therapy, median OS was 11.9 (95% confidence interval 5.1–16.2), 11.1 (6.9–16.7) and 8.1 (5.2–11.4) months, respectively, and median PFS was 4.4 (2.8–6.5), 4.3 (2.9–6.3) and 3.4 (2.2–5.2) months, respectively. Common adverse events (AEs) (any grade) included gastrointestinal disorders (64.7%) and asthenia/fatigue (63.3%). In routine clinical practice, ziv-aflibercept was frequently initiated in third line or later lines of therapy. Although patients receiving ziv-aflibercept were more heavily pretreated and potentially less robust compared with the VELOUR trial, median OS for patients receiving second-line ziv-aflibercept was comparable. AE rates were similar to or lower than the VELOUR trial.

## Introduction

Colorectal cancer (CRC) is the fourth most common cancer in the USA, with 132,700 new cases and 49,700 deaths estimated to occur in 2015 [1]. One-quarter of CRC patients present with metastases at diagnosis, and nearly 50% will develop metastases during the course of their disease [2]. While the estimated CRC-related 5-year survival rate approaches 60% overall, 5-year survival for metastatic disease (mCRC) remains only 12% [2, 3].

Systemic therapy remains the principal treatment option for mCRC. Chemotherapies used to treat mCRC are commonly based on fluoropyrimidines, combining infusional fluorouracil and leucovorin with either oxaliplatin (FOLFOX regimen) or irinotecan (FOLFIRI regimen) [4]. Several studies have evaluated vascular endothelial growth factor (VEGF)-targeted agents such as bevacizumab, ziv-aflibercept and ramucirumab in combination with chemotherapy and have reported statistically significant improvements in overall survival (OS) for patients with mCRC. A phase 3 trial demonstrated an OS and progression-free survival (PFS) benefit with bevacizumab when added to irinotecan, fluorouracil and leucovorin (IFL) compared with IFL plus placebo in previously untreated patients with mCRC [5]. In addition, the phase 3 ‘RAISE’ trial demonstrated an OS benefit with ramucirumab plus FOLFIRI compared with FOLFIRI plus placebo in patients with mCRC with disease progression during or after first-line therapy with bevacizumab, oxaliplatin and a fluoropyrimidine [6].

The randomized, double-blind, phase 3 ‘VELOUR’ trial evaluated the efficacy and safety of ziv-aflibercept plus FOLFIRI as second-line treatment for patients with mCRC who had disease progression on or after oxaliplatin-based treatment [7]. Prior use of bevacizumab was permitted, but all patients were irinotecan-naïve. Compared with placebo plus FOLFIRI, the addition of ziv-aflibercept to FOLFIRI significantly improved both OS (13.5 vs. 12.1 months; hazard ratio [HR] 0.82; *p* = 0.0032) and PFS (6.9 vs. 4.7 months; HR 0.76; *p* < 0.0001). Anti-VEGF treatment-related toxicities such as hypertension, mucosal bleeding and proteinuria were reported in more patients in the ziv-aflibercept arm compared with the control arm.

As clinical trials often include a select patient population, it remains uncertain whether the results of a new agent in a clinical trial can be replicated in routine ‘real-world’ clinical practice. To date, no studies have reported treatment patterns and effectiveness in real-world clinical practice among patients with mCRC treated with ziv-aflibercept. The objectives of this study were to assess real-world characteristics, treatment patterns, effectiveness and adverse events (AEs) in patients with mCRC who have initiated ziv-aflibercept treatment as second-line or later-line therapy following a prior oxaliplatin-based regimen.

## Methods

### Study design

This retrospective cohort study aimed to assess patients with mCRC included in the Vector Oncology Data Warehouse and Altos Solutions Inc. Database who had initiated ziv-aflibercept as second-line or later-line therapy following an oxaliplatin-based treatment. The databases are comprehensive cancer patient databases derived from networks of community oncology practices in the USA, comprising demographic information, medical history, treatment information, laboratory results and clinical information from electronic medical records (EMRs) and billing data. This study included review of de-identified structured EMR data (from Jan 2003 to Jul 2014) and a manual review of unstructured preexisting EMR data (such as physician notes and EMR attachments) for fields that are not well populated in the structured EMR dataset [Health Insurance Portability and Accountability Act (HIPAA) compliant data were collected]. The study protocol was reviewed and approved by the New England Independent Review Board. Because of the retrospective nature of this study, patient informed consent was not required in accordance with institutional regulations.

### Inclusion criteria

Eligible patients had mCRC and had received ziv-aflibercept (second line or later) not as part of a clinical trial. Patients were required to have received oxaliplatin therapy prior to ziv-aflibercept initiation. In addition, patients were required to have no other primary tumours during the baseline period (1-year period before ziv-aflibercept initiation).

### Outcomes measures

The date of ziv-aflibercept initiation was defined as the index date. The follow-up period started at the date of ziv-aflibercept initiation and ended at the data cut-off date, last clinic visit date or death. Baseline clinical characteristics were assessed before or at the index date of ziv-aflibercept initiation, and the distribution of the line of ziv-aflibercept initiation was described. Lines of therapy after mCRC diagnosis were identified using the following algorithm based on chemotherapy/targeted therapy administrations. The start of first-line therapy was defined as the first administration of chemotherapy or targeted therapy after diagnosis of mCRC. The first-line regimen contained all chemotherapy-related drugs administered within 28 days of the start of the regimen. Subsequent lines of therapy were identified by the first administration of any chemotherapy-related drug not used in the previous line of therapy. The subsequent regimens contained all chemotherapy-related drugs that were administered within 28 days of the first administration in that line of therapy. If there was a gap of ≥ 120 days between treatment administrations within a given line of therapy, the second treatment was considered a new line of therapy. Chemotherapy or targeted therapy administrations were identified based on review of generic names of administered medications, review of generic names for oral medication prescriptions and information for oral chemotherapy medications. The end date for each line of therapy was defined as the last date of administration of any of the drugs used in the regimen before the start of the subsequent line of therapy. Duration of ziv-aflibercept treatment was measured in days from the first administration of ziv-aflibercept to the last administration of ziv-aflibercept. Duration of the chemotherapy regimen containing ziv-aflibercept was measured in days from the first administration of the first drug administered in the regimen to the last administration of any of the chemotherapy drugs used in the regimen before the start of the subsequent line of therapy. Patients were considered censored if the last administration of either the ziv-aflibercept or the concomitant chemotherapy regimen was within 60 days of the date of last contact. Reasons behind discontinuation of ziv-aflibercept and the concomitant regimen, reasons for treatment interruptions and dose changes, and the date of last contact/death were also collected.

Other outcomes data (effectiveness, all treatment-emergent AEs and resource utilization) were also assessed. Objective tumour response was based on information available in the EMR notes; to the extent information was available, tumour response was further classified as complete or partial. OS was defined as the time from ziv-aflibercept initiation to death or last contact (for censored patients). PFS was defined as the time from ziv-aflibercept initiation to first disease progression or death (whichever occurred earliest) or last contact (for censored patients). Information about treatment-emergent AEs during ziv-aflibercept treatment was extracted for prespecified events, including those reported in the ziv-aflibercept prescribing information. For resource utilization, the number of patients with hospitalizations and emergency room visits after ziv-aflibercept initiation was reported.

### Statistical analyses

Statistical analysis was performed on the Altos Solutions and Vector Oncology datasets, both individually and on the pooled data. Data extracted from the individual databases are reported for baseline patient and clinical characteristics in this publication; remaining data and analyses are shown for the pooled dataset.

To assess the heterogeneity of the populations from the two data sources, meta-analysis random effects models were estimated for select baseline characteristics, including age, gender, race, tumour location, number of sites of metastases, occurrence of liver metastases, presence of hypertension, months from first mCRC diagnosis to ziv-aflibercept initiation, and prior bevacizumab use [8].

Continuous variables were summarized by mean, median, standard deviation and interquartile ranges (or minimum and maximum). Categorical variables were summarized by absolute frequencies and percentages. Kaplan–Meier analyses were performed to evaluate time to discontinuation of the entire chemotherapy regimen concomitant with ziv-aflibercept, time to ziv-aflibercept discontinuation, PFS and OS. Survival rates at 6 and 12 months were also reported. These variables were analysed both in the overall population and according to the line of therapy at which ziv-aflibercept was initiated. All data manipulations and statistical analyses were performed using SAS version 9.3 software.

## Results

### Baseline patient and disease characteristics

In total, 218 patients were included in the final study population (Vector Oncology database, *n* = 107; Altos Solutions database, *n* = 111). The mean age at ziv-aflibercept initiation was 62.8 years (Table 1). The majority of patients were male (61.0%) and Caucasian (59.6%). Notably, the majority of the patient population (93.5%) identified from the Vector Oncology database were geographically located in the South of the USA, while the patient population identified from the Altos Solutions database was more evenly distributed among the Northeast (30.6%), South (30.6%) and West (23.4%). Heterogeneity tests found no significant (*p* < 0.05) heterogeneity between populations for most baseline characteristics except months from mCRC diagnosis to ziv-aflibercept initiation and prior bevacizumab use.

**Table 1:** Baseline patient and clinical characteristics (baseline characteristics were assessed before or at the index date of ziv-aflibercept initiation) and ziv-aflibercept treatment characteristics

	Vector Oncology (*N* = 107)	Altos Solutions (*N* = 111)	Pooled data (*N* = 218)
*Baseline patient and clinical characteristics*			
Age at ziv-aflibercept initiation (years)			
Mean (SD)	62.6 (10.4)	63.0 (12.1)	62.8 (11.2)
Median (IQR)	63.0 (55, 70)	62.0 (55, 71)	63.0 (55, 71)
Age group in years at ziv-aflibercept initiation, *n* (%)			
18–64	59 (55.1)	61 (55.0)	120 (55.0)
65–74	32 (29.9)	30 (27.0)	62 (28.4)
≥ 75	16 (15.0)	20 (18.0)	36 (16.5)
Male, *n* (%)	67 (62.6)	66 (59.5)	133 (61.0)
Race, *n* (%)			
Caucasian	54 (50.5)	76 (68.5)	130 (59.6)
African American	21 (19.6)	7 (6.3)	28 (12.8)
Other	17 (15.9)	23 (20.7)	40 (18.3)
Unknown	15 (14.0)	5 (4.5)	20 (9.2)
Geographic USA region^a^, *n* (%)			
Northeast	0 (0.0)	34 (30.6)	34 (15.6)
South	100 (93.5)	34 (30.6)	134 (61.5)
West	3 (2.8)	26 (23.4)	29 (13.3)
Midwest	3 (2.8)	16 (14.4)	19 (8.7)
Other	0 (0.0)	1 (0.9)	1 (0.5)
Unknown	1 (0.9)	0 (0.0)	1 (0.5)
Time from mCRC diagnosis to ziv-aflibercept initiation (months)			
Mean (SD)	29 (22.2)	22 (14.0)	25 (18.8)
Median (IQR)	21 (14, 35)	18 (12, 28)	20 (13, 30)
Location of primary tumour, *n* (%)			
Colon	89 (83.2)	82 (73.9)	171 (78.4)
Rectum	18 (16.8)	27 (24.3)	45 (20.6)
Unknown	0 (0.0)	2 (1.8)	2 (0.9)
ECOG PS prior to ziv-aflibercept initiation, *n* (%)			
0	31 (29.0)	20 (18.0)	51 (23.4)
1	50 (46.7)	31 (27.9)	81 (37.2)
2	15 (14.0)	7 (6.3)	22 (10.1)
≥ 3	3 (2.8)	0 (0.0)	3 (1.4)
Unknown	8 (7.5)	53 (47.7)	61 (28.0)
Sites of metastasis at ziv-aflibercept initiation, *n* (%)			
Liver	88 (82.2)	85 (76.6)	173 (79.4)
Lung	58 (54.2)	60 (54.1)	118 (54.1)
Lymph nodes	20 (18.7)	51 (45.9)	71 (32.6)
Peritoneum	13 (12.1)	16 (14.4)	29 (13.3)
Other	39 (36.4)	27 (24.3)	66 (30.3)
Number of metastatic sites at ziv-aflibercept initiation^b^, *n* (%)			
1	34 (31.8)	27 (24.3)	61 (28.0)
2	32 (29.9)	39 (35.1)	71 (32.6)
≥ 3	36 (33.6)	40 (36.0)	76 (34.9)
Missing	5 (4.7)	5 (4.5)	10 (4.6)
Common conditions diagnosed prior to treatment with ziv-aflibercept, *n* (%)			
Hypertension	64 (59.8)	57 (51.4)	121 (55.5)
Tobacco user	44 (41.1)	21 (18.9)	65 (29.8)
Diabetes mellitus	18 (16.8)	12 (10.8)	30 (13.8)
Dyslipidaemia	11 (10.3)	19 (17.1)	30 (13.8)
Venous thromboembolic event	11 (10.3)	9 (8.1)	20 (9.2)
Arterial thromboembolic event	8 (7.5)	6 (5.4)	14 (6.4)
Renal failure	9 (8.4)	2 (1.8)	11 (5.0)
Patients treated with bevacizumab for mCRC prior to ziv-aflibercept initiation, *n* (%)	105 (98.1)	95 (85.6)	200 (91.7)
Patients treated with irinotecan for mCRC prior to ziv-aflibercept initiation, *n* (%)	72 (67.3)	58 (52.3)	130 (59.6)

	Pooled data (*N* = 218)
*Ziv-aflibercept treatment characteristics*	
Line of mCRC treatment in which ziv-aflibercept was initiated, *n* (%)	
Second line	54 (24.8)
Third line	69 (31.7)
Fourth line	47 (21.6)
Fifth line	26 (11.9)
Sixth line	16 (7.3)
Seventh line	3 (1.4)
Eighth line	1 (0.5)
Eleventh line	1 (0.5)
Thirteenth line	1 (0.5)
Initial dose of ziv-aflibercept, mg/kg	
Mean (SD)^c^	3.9 (0.6)
Median (IQR)	4 (4, 4)
Length of available follow-up after initiation of chemotherapy regimen containing ziv-aflibercept, months	
Mean (SD)	8.8 (6.6)
Median (IQR)	7.1 (3.2, 13.9)
Concomitant chemotherapy regimen (> 1% of patients), *n* (%)	
FOLFIRI	195 (89.4)
Irinotecan	11 (5.0)
Fluorouracil	5 (2.3)
Others	3 (1.5)
None	4 (1.8)
Time to discontinuation of entire chemotherapy regimen containing ziv-aflibercept	
Mean, months (SE)	6.2 (0.5)
Median, months (95% CI)	4.5 (2.8, 5.3)
Reasons, *n* (%)	
Disease progression	91 (53.5)
AE/toxicity	26 (15.3)
Patient’s decision	19 (11.2)
Physician’s decision	7 (4.1)
Other^d^	12 (7.1)
Unknown	13 (7.6)
Missing	1 (0.6)
Time to discontinuation of ziv-aflibercept	
Mean, months (SE)	5.3 (0.4)
Median, months (95% CI)	4.6 (2.8, 5.1)
Reasons, *n* (%)	
Disease progression	94 (49.0)
Adverse event/toxicity	45 (23.4)
Patient’s decision	16 (8.3)
Physician’s decision	7 (3.6)
Other^e^	15 (7.8)
Unknown	15 (7.8)
Missing	0
Median time to discontinuation of ziv-aflibercept by line of initiation, months (IQR)	
Second line	4.9 (2.1, 6.0)
Third line	5.1 (2.8, 7.0)
Fourth line	3.0 (1.9, 4.6)

*AE* adverse event, *CI* confidence interval, *ECOG PS* Eastern Cooperative Oncology Group Performance Status, *IQR* interquartile range, *mCRC* metastatic colorectal cancer, *SD* standard deviation, *SE* standard error

^a^Geographic regions as defined by the US Census Bureau [11]

^b^Lymph nodes were considered to be one site

^c^The weight measured on the date closest to the initial dosage date was used. For Altos Solutions, one patient did not have any recorded weight information and was excluded from the calculation of dose

^d^Other reasons included death, insurance refused coverage, patient hospitalized, patient moved and patient admitted to nursing home with comfort care

^e^Other reasons included death, patient moved and patient admitted to nursing home with comfort care

In patients with available biomarker data for KRAS (*n* = 179), 46.4% had wildtype KRAS and 53.6% had a KRAS gene mutation; among patients with biomarker data for BRAF (*n* = 46), 82.6% had wildtype BRAF and 17.4% had a BRAF gene mutation. The most common site of metastasis was the liver (79.4%). The most frequent conditions diagnosed prior to ziv-aflibercept initiation were hypertension (55.5%) and tobacco use (29.8%).

### Treatment patterns

**Fig. 1:**
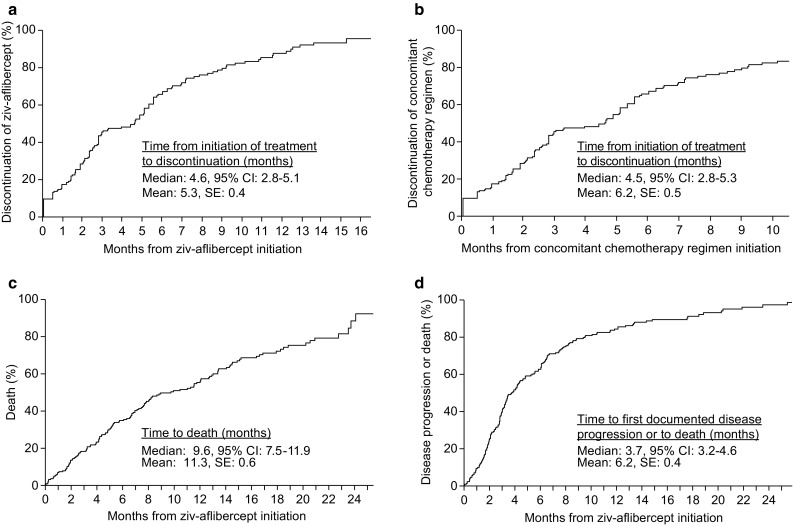
Kaplan–Meier estimates for **a** time to discontinuation of ziv-aflibercept, **b** time to discontinuation of concomitant chemotherapy regimen, **c** overall survival and **d** progression-free survival. *CI* confidence interval, *SE* standard error

Overall, 24.8% of patients initiated ziv-aflibercept as second-line therapy for mCRC (Table 1). The majority of patients initiated ziv-aflibercept treatment in the third-line or later-line setting, with 31.7% of patients receiving ziv-aflibercept as third-line therapy, 21.6% as fourth-line therapy, 11.9% as fifth-line therapy and 10.1% of patients initiating ziv-aflibercept beyond the fifth-line setting. The median initial dose of ziv-aflibercept in 2-week treatment cycles was 4 mg/kg. Dose adjustments of ziv-aflibercept occurred in 9.2% of patients, mainly resulting from AEs/toxicity (80.0%). The majority of patients received bevacizumab (91.7%) or irinotecan (59.6%) as part of therapy for mCRC and prior to ziv-aflibercept initiation. In total, 90.4% of patients initiated ziv-aflibercept with FOLFIRI, of which 48.7% had previously received FOLFIRI. Among patients initiating ziv-aflibercept with irinotecan (95.4%), 59.6% of patients had previously received irinotecan. After initiation of ziv-aflibercept, the median length of follow-up was 7.1 months. The median duration of treatment with ziv-aflibercept was 4.6 months (Table 1 and Fig. 1a). The median duration of treatment with the first chemotherapy regimen including ziv-aflibercept was 4.5 months (Table 1 and Fig. 1b). The median duration of ziv-aflibercept treatment was slightly longer among patients who initiated ziv-aflibercept in second-line therapy (4.9 months; 95% confidence interval [CI] 2.1–6.0) or in third-line therapy (5.1 months; 95% CI 2.8–7.0) relative to the overall population. The most frequent reasons for discontinuation of ziv-aflibercept were disease progression (49.0%) and AEs/toxicity (23.4%).

### Effectiveness analysis

Effectiveness among patients treated with ziv-aflibercept in routine clinical practice was evaluated in terms of best tumour response, PFS and OS (Fig. 1c, d and Table 2). Overall, 21.1% of patients achieved objective response, and 17.4% achieved stable disease as best tumour response after initiation of ziv-aflibercept. In patients initiating ziv-aflibercept as second-line (*n* = 54), third-line (*n* = 69) and fourth-line (*n* = 47) treatment, 25.9, 20.3 and 21.3% achieved complete or partial response, respectively, and 14.8, 21.7 and 19.1% achieved stable disease.

**Table 2:** Ziv-aflibercept effectiveness analysis

	Pooled population	By line of therapy at ziv-aflibercept initiation		
	All lines (*N* = 218)	Second line (*n* = 54)	Third line (*n* = 69)	Fourth line (*n* = 47)
Response^a^				
Best tumour response following ziv-aflibercept initiation, *n* (%)				
Objective response	46 (21.1)	14 (25.9)	14 (20.3)	10 (21.3)
Stable disease	38 (17.4)	8 (14.8)	15 (21.7)	9 (19.1)
Progressive disease	50 (22.9)	12 (22.2)	12 (17.4)	14 (29.8)
Unknown	62 (28.4)	13 (24.1)	23 (33.3)	12 (25.5)
Not evaluable	22 (10.1)	7 (13.0)	5 (7.2)	2 (4.3)
Overall survival				
Survival rates (KM estimates), % (95% CI)				
6 months after treatment initiation	65.1 (58.1, 71.2)	–	–	–
12 months after treatment initiation	42.7 (35.4, 49.7)	–	–	–
Time to death, months				
Median (95% CI)	9.6 (7.5, 11.9)	11.9 (5.1, 16.2)	11.1 (6.9, 16.7)	8.1 (5.2, 11.4)
Progression-free survival				
Survival rates (KM estimates), % (95% CI)				
6 months after treatment initiation	36.2 (29.7, 42.8)	–	–	–
12 months after treatment initiation	14.4 (9.8, 19.9)	–	–	–
Median time to disease progression or death, months (95% CI)	3.7 (3.2, 4.6)	4.4 (2.8, 6.5)	4.3 (2.9, 6.3)	3.4 (2.2, 5.2)

*CI* confidence interval, *CT* computerized tomography, *KM* Kaplan–Meier

^a^Best tumour response was based on radiology assessment of CT scan from chart review

The estimated 6-month OS rate following ziv-aflibercept initiation was 65.1% (95% CI 58.1–71.2) and the estimated 12-month OS rate was 42.7% (95% CI 35.4–49.7). At the end of study follow-up, 35.3% of patients remained alive and the median OS was 9.6 months (95% CI 7.5–11.9) (Fig. 1c). In patients who initiated ziv-aflibercept as second-line, third-line and fourth-line therapy, median OS was 11.9 months (95% CI 5.1–16.2), 11.1 months (95% CI 6.9–16.7) and 8.1 months (95% CI 5.2–11.4), respectively. The estimated 6-month PFS rate following ziv-aflibercept initiation was 36.2% (95% CI 29.7–42.8) and the estimated 12-month PFS rate was 14.4% (95% CI 9.8–19.9). The median PFS was 3.7 months (95% CI 3.2–4.6) (Fig. 1d), and 190 of 218 patients (87.2%) experienced disease progression or death. In patients who initiated ziv-aflibercept as second-line, third-line and fourth-line therapy, median PFS was 4.4 months (95% CI 2.8–6.5), 4.3 months (95% CI 2.9–6.3) and 3.4 months (95% CI 2.2–5.2), respectively.

### Symptom burden and healthcare resource utilization

The most frequent treatment-emergent AEs occurring in > 20% of patients during treatment with ziv-aflibercept were gastrointestinal disorders (64.7%), asthenia/fatigue (63.3%), thrombocytopenia (27.1%), neutropenia (25.7%), hypertension (24.3%), infection (23.4%) and decreased appetite (22.0%) (Table 3). The most frequent Grade ≥ 3 AEs occurring in > 5% of patients were gastrointestinal disorders (11.0%), asthenia/fatigue (8.7%), neutropenia (8.7%) and hypertension (6.4%). Regarding healthcare resource utilization after initiation of ziv-aflibercept, 31.2% of patients had at least one hospitalization and 16.1% had at least one emergency room visit by the end of study follow-up.

**Table 3:** Treatment-emergent AEs experienced during treatment with ziv-aflibercept (all-grade AEs occurring in > 5% of the total population)

AEs	All grades, *n* (%)	Grade ≥ 3^a^ *n* (%)	Mean number of cycles AE experienced, *n*
Gastrointestinal disorders (e.g. diarrhoea, stomatitis, abdominal pain)	141 (64.7)	24 (11.0)	3.5
Asthenia/fatigue	138 (63.3)	19 (8.7)	3.6
Thrombocytopenia	59 (27.1)	7 (3.2)	3.1
Neutropenia	56 (25.7)	19 (8.7)	2.3
Hypertension	53 (24.3)	14 (6.4)	3.1
Infection	51 (23.4)	9 (4.1)	1.2
Decreased appetite	48 (22.0)	1 (0.5)	3.0
Weight decrease (at least 5%)	48 (22.0)	1 (0.5)	2.7
Dehydration	36 (16.5)	10 (4.6)	2.1
Leukopenia	34 (15.6)	10 (4.6)	2.0
Haemorrhage	33 (15.1)	3 (1.4)	1.5
Generalized pain	31 (14.2)	6 (2.8)	1.9
Proteinuria	31 (14.2)	5 (2.3)	1.8
AST increase	33 (15.1)	4 (1.8)	2.4
Nausea	26 (11.9)	4 (1.8)	2.4
ALT increase	24 (11.0)	2 (0.9)	1.5
Anaemia	20 (9.2)	2 (0.9)	2.4
Serum creatinine increase	17 (7.8)	0	1.9
Neuropathy	13 (6.0)	1 (0.5)	2.5

*AE* adverse event, *ALT* alanine aminotransferase, *AST* aspartate aminotransferase

^a^Severity grades for AEs were based on National Cancer Institute Common Terminology Criteria for Adverse Events v4.0 [12]

## Discussion

Randomized clinical trials are essential for establishing the efficacy and safety of new drugs, and for informing regulatory approval and clinical practice algorithms. Nonetheless, clinical trials often enrol a narrowly defined patient population who are treated under highly controlled parameters. As such, studies that examine the use, effectiveness and safety of interventions in patients treated outside of clinical trials, in the context of routine clinical practice, can further inform the practical utility of a new agent.

This retrospective analysis studied patients with mCRC who received ziv-aflibercept as second-line or later-line therapy following a prior oxaliplatin-based treatment regimen. In this sample of USA patients treated with ziv-aflibercept outside of a clinical trial and within non-academic community oncology practices, treatment characteristics differed from patients in the phase 3 trial of ziv-aflibercept plus FOLFIRI as second-line treatment in patients with mCRC (VELOUR). In the VELOUR study, ziv-aflibercept was administered as part of second-line treatment in all patients. In contrast, in this real-world analysis only 24.8% of patients initiated ziv-aflibercept as second-line therapy, more than two-thirds of patients initiated ziv-aflibercept as third-line or later-line therapy, and six patients (2.9%) initiated ziv-aflibercept in the seventh line or later. Also, nearly 60% of patients in this analysis received prior irinotecan-based therapy compared with the VELOUR trial, which required patients to be irinotecan-naïve. In addition, over 90% of patients in this analysis received bevacizumab prior to the initiation of ziv-aflibercept, compared with only 30% of patients enrolled in the VELOUR trial [7].

Despite patients in this analysis being more heavily pretreated—and potentially less physically fit—compared with the VELOUR trial, the median OS for patients receiving second-line ziv-aflibercept was approximately 12 months for both studies [7]. The median OS was also comparable to other previously published trials of second-line anti-VEGF therapy in patients with mCRC, including the ECOG Study E3200 (FOLFOX4 plus bevacizumab in mCRC; median OS 11.9 months) and the RAISE trial (ramucirumab plus FOLFIRI in mCRC; 13.3 months) [6, 9]. In this analysis, approximately 20% of all patients responded to treatment. Almost 26% of patients responded to second-line treatment, which is comparable with both the VELOUR (19.8%) and ECOG E3200 (22.7%) studies [6, 9].

The rate of treatment-emergent AEs experienced by patients in this analysis was similar to or lower than the rates observed in the VELOUR trial. In the VELOUR trial, the most commonly experienced AEs were anaemia (82.3%; Grade ≥ 3: 3.8%), diarrhoea (69.2%; Grade ≥ 3: 19.3%), neutropenia (67.8%; Grade ≥ 3: 36.7%), proteinuria (62.2%; Grade ≥ 3: 7.8%) and asthenic conditions (60.4%; Grade ≥ 3: 16.8%) [7]. In this real-world analysis, the most common AEs were gastrointestinal disorders (e.g. diarrhoea, stomatitis, abdominal pain, 64.7%; Grade ≥ 3: 11.0%) and asthenia/fatigue (63.3%; Grade ≥ 3: 8.7%). Anaemia was reported in only 9.2% of patients, neutropenia in only 25.7% and proteinuria in only 14.2% of patients.

We acknowledge that these findings should be interpreted with caution; data generated from an analysis of patients treated in routine clinical practice and from clinical trials cannot be directly compared because of differences in patient populations. As a study using EMR data, this analysis has several limitations. Data fields such as clinical characteristics are not required for reimbursement and hence are often not well populated. Also, prescription data (e.g. for oral chemotherapy agents) are not as reliable as administration data for injectable/infused chemotherapy agents. To mitigate these concerns and obtain more complete and accurate data, this study supplemented the structured EMR data with a review of unstructured EMR records from the Altos Solutions and Vector Oncology databases. Also, the determination of chemotherapy regimens and lines of therapy from individual administrations of chemotherapy and targeted agents is sensitive to the specific algorithm that is used. Therefore, an algorithm with different rules may yield different results. In addition, the overall sample size and follow-up for this study was limited by the relatively recent approval of ziv-aflibercept in 2012 [10]. Treatment-emergent AE data were obtained to the extent that they were recorded in the EMR records and may underestimate the overall rate of AEs experienced by the patients. Finally, stratified effectiveness analyses by line of therapy at initiation of ziv-aflibercept were not adjusted for patient characteristics.

In summary, this study evaluated routine clinical practice data of patients with mCRC who initiated ziv-aflibercept as second-line or later-line therapy following a prior oxaliplatin-based treatment regimen. Ziv-aflibercept was frequently initiated in the third-line or later-line setting during the first 2 years after drug approval in the USA. Although patients treated with ziv-aflibercept in our cohort were typically more heavily pretreated, and potentially less robust, than in the VELOUR clinical trial, the median OS was similar to VELOUR and also to other previously published trials of second-line anti-VEGF therapy in mCRC [6, 7, 9]. The rate of AEs experienced by patients in this analysis was similar to or lower than the VELOUR trial [7].
